# Determining the toxicological effects of indoor air pollution on both a healthy and an inflammatory-comprised model of the alveolar epithelial barrier in vitro

**DOI:** 10.1186/s12989-024-00584-8

**Published:** 2024-05-17

**Authors:** Kirsty Meldrum, Stephen J. Evans, Michael J. Burgum, Shareen H. Doak, Martin J. D. Clift

**Affiliations:** In Vitro Toxicology Group, Swansea University Medical School, Swansea University, Singleton Park Campus, Swansea, Wales SA2 8PP UK

**Keywords:** Indoor air pollution, In vitro, Particulate matter, Inhalation, Lung, Disease model, Healthy

## Abstract

Exposure to indoor air pollutants (IAP) has increased recently, with people spending more time indoors (*i.e.* homes, offices, schools and transportation). Increased exposures of IAP on a healthy population are poorly understood, and those with allergic respiratory conditions even less so. The objective of this study, therefore, was to implement a well-characterised in vitro model of the human alveolar epithelial barrier (A549 + PMA differentiated THP-1 incubated with and without IL-13, IL-5 and IL-4) to determine the effects of a standardised indoor particulate (NIST 2583) on both a healthy lung model and one modelling a type-II (stimulated with IL-13, IL-5 and IL-4) inflammatory response (such as asthma).

Using concentrations from the literature, and an environmentally appropriate exposure we investigated 232, 464 and 608ng/cm^2^ of NIST 2583 respectively. Membrane integrity (blue dextran), viability (trypan blue), genotoxicity (micronucleus (Mn) assay) and (pro-)/(anti-)inflammatory effects (IL-6, IL-8, IL-33, IL-10) were then assessed 24 h post exposure to both models. Models were exposed using a physiologically relevant aerosolisation method (VitroCell Cloud 12 exposure system).

No changes in Mn frequency or membrane integrity in either model were noted when exposed to any of the tested concentrations of NIST 2583. A significant decrease (*p* < 0.05) in cell viability at the highest concentration was observed in the healthy model. Whilst cell viability in the “inflamed” model was decreased at the lower concentrations (significantly (*p* < 0.05) after 464ng/cm^2^). A significant reduction (*p* < 0.05) in IL-10 and a significant increase in IL-33 was seen after 24 h exposure to NIST 2583 (464, 608ng/cm^2^) in the “inflamed” model.

Collectively, the results indicate the potential for IAP to cause the onset of a type II response as well as exacerbating pre-existing allergic conditions. Furthermore, the data imposes the importance of considering unhealthy individuals when investigating the potential health effects of IAP. It also highlights that even in a healthy population these particles have the potential to induce this type II response and initiate an immune response following exposure to IAP.

**Supplementary Information:**

The online version contains supplementary material available at 10.1186/s12989-024-00584-8.

## Background

Air pollution is not a new problem but a gradually changing one. One thing that has, without a doubt changed is the attitude towards pollution [1].

Pollution, both outdoor and indoor is made up of various components depending on the source(s). Recently, the impact of air pollution has been brought back to the public’s attention with the publication of the Chief Medical Officer’s annual report 2022 [2] after outdoor air pollution was categorised as *carcinogenic to humans* (Group 1) by the International Agency for Research on Cancer (IARC) [3]. Pollution can be compromised of particulate matter, gaseous compounds, volatile organic (and chemical) compounds [4] and biological components (bacteria, viruses, fungi and various allergens) [5]. Particles in both outdoor and indoor environments are a mixture of sizes and can be categorised based on their aerodynamic diameter; course particles (PM_10_, 2.5-10µm); fine particles (PM_2.5_, < 2.5µm); and ultrafine particles (UF, PM 0.1, < 100nm) [6, 7]. Health effects associated with indoor air pollution (IAP) are linked to reduced air quality within the indoor environment as exposure is low and sustained. This includes Sick Building Syndrome (SBS), Building Related Illness (BRI) and respiratory infections and cardiovascular diseases [8].

IAP risks are becoming more apparent as the public and toxicological focus shifts from outdoor air pollution. A study published over 20 years ago [9] concluded that the average person spends around 87% of their time in enclosed buildings. This was before the boom in technology such as smart phones, game consoles and working from home (a propensity enhanced by the lockdowns associated with the SARS-COV-2 epidemic). Thus, it is highly possible that we are now spending much more time indoors than 20 years ago. Indoor air pollution doesn’t just infer homes, it encompasses transportation (buses, trains and cars) as well as education (schools, nurseries, and universities) and work and social places (offices, hospitals, supermarkets and gyms); differentiating into private indoor, and public indoor environments [2]. There is, therefore, the potential for indoor pollution exposure to increase due to greater time spent indoors, different environments, the age of the building and the type of housing stock [10, 11].

The composition of indoor air pollution can be influenced by several different factors. These include the presence of carpets [12], the time of the year [13], the level of dampness [14], the microbiome of the person in that environment [15, 16], the use of indoor space [17], socio-economic status [18, 19], fuel use [20], what the area is used for (*e.g.,* cooking [21, 22], cleaning, personal hygiene [23], burning candles [24]), and even the boundary points with the outside environment (*e.g*., open windows) [25].

The main route of exposure to all indoor air pollution components is via inhalation and therefore it is important to investigate the impacts within the airways. PM_2.5_ is known to deposit within the alveolar region, and therefore this region is of particular interest [26]. A utilised in vitro model for this region of the airway is an A549 + THP-1 co-culture at the air–liquid interface (ALI). This model is used due to the characteristics of the A549 cells and their similarity to alveolar epithelial type II cells (ATII) and the availability of the THP-1 cells to be differentiated to macrophage like cells instead of implementing whole blood isolation of macrophages. Type II alveolar cells have been identified as having major histocompatibility complex class II (MHCII) receptors. This complex is important in activating the adaptive immune repose and indicates that Type II cells have the potential to influence the adaptive immune response when exposed to various antigens [27]*, e.g.,* MHC-II presents to CD4^+^ T cells [28].

The addition of immune cells to an epithelial cell culture is well documented to increase the responses of the model and reflect the physiological conditions of the alveolar region of the lung closer than a monoculture [29–31]. The exposure conditions of such a model are also relevant and have been previously shown to influence the biological responses of the model [32].

A link has been identified between the concentration of deposited PM_2.5_ and the development of emphysema in COPD patients [33], as well as the association of PM with various respiratory diseases (*i.e.,* bronchial asthma, development of lung cancer, idiopathic pulmonary fibrosis and pneumonia) and increases in mortality related to these pulmonary illnesses [34]. With most in vitro studies completed on an assumed “healthy” model, the fact that PM has been linked to lung diseases and mortality, highlights the importance of also determining the effects these particulates may have on an “inflamed” lung model or one that is representative of an airway disease (*e.g.,* susceptible group of the population). Chronic inflammatory diseases are driven by type-II inflammation including both innate lymphoid cells (ILC2) and type 2 T-helper cells (Th2). Both cell types produce IL-4, IL-5 and IL-13 [35] leading to an inflammatory cascade and the development of inflammatory disease(s) [36]. These type-II inflammatory mediators along with TSLP and IgE remain a target for biological therapies for these diseases [37]. Alveolar inflammation has been identified as being increased in patients with asthma, influencing the disease and the immune cells that are present within the alveolar region [38]. The influence of PM has mostly been investigated in murine models and it is important to further understanding of these diseases in humans, and human-relevant models [39]. This is due to the fact that some of these conditions and disease states are human specific (therefore have to be induced within animal models). It is important that the potential impacts exposures to environmental air pollutants (with emphasis on indoor PM) may have on the public are investigated with chronic inflammatory diseases of the airways compared to a healthy population.

Thus, the overarching aim of this study was to expose a previously established model of the alveolar region of the lung to a standardised indoor air pollution particulate (NIST), as well as to further this exposure by developing and characterising an “immune” model of the same area and comparing the endpoints—developing an “allergic” phenotype. The objective of this study was to determine if the addition of inflammatory mediators to an established and characterised co-culture model had the potential to influence the biological outputs. This was completed by implementing a physiologically relevant exposure method as well a standard particulate as a surrogate for collected particles. It was hypothesised that the “immune” model would have an increased response to the exposure of the indoor air pollution particles.

## Materials and methods

**Fig. 1:**
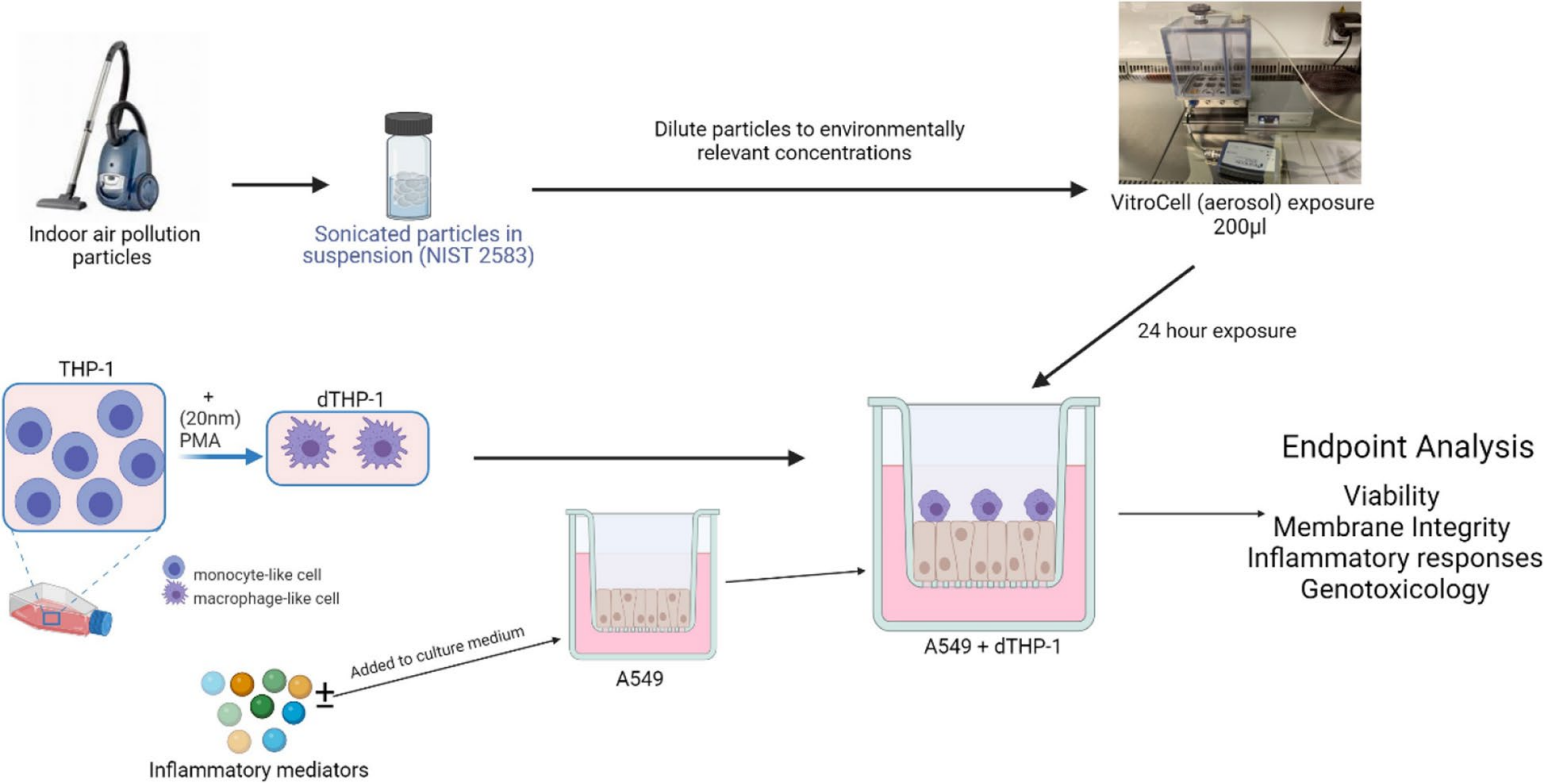
Experimental approach conducted within this study. An exposure to the A549 + d-THP-1 co-culture of indoor air pollution particles (NIST 2583) is completed and analysed 24 h after exposure via the VitroCell (aerosol) exposure method. Created with BioRender.com

All chemicals and reagents were purchased from Sigma Aldrich (UK) unless otherwise stated. The various exposures and models implemented is outlined in Fig. 1.

### Cell cultures

A549 (ATCC® CCL-185™) cells were obtained from American Tissue Culture Collection (ATCC, USA) and were cultured at 37°C in 5% CO_2_. A549 were cultivated in RPMI-1640 medium (Gibco, USA) supplemented with 10% heat inactivated foetal bovine serum (FBS, Gibco, USA), 2mM L-Glutamine (Gibco, USA), 100U/mL penicillin and 100µg/mL streptomycin (Gibco, USA), cited as complete cell culture medium (CCM). Cells were passaged when ~ 80% confluent and used between passages 15–21 for all experimentation [40].

THP-1 (ATCC® TIB-202™) cells were obtained from American Tissue Culture Collection (ATCC, USA) and were cultured at 37°C in 5% CO_2_. THP-1 were cultivated in the above CCM. Cells were maintained within cultures of 1 × 10^6^ cells/ml and used between passages 10–15 for all experimentation.

The co-culture was seeded and switched to the air–liquid interface (ALI) as previously outlined [32]. Briefly, A549 cells were seeded at a density of 2.78 × 10^5^ cells/cm^2^ on the apical side of a Falcon 12 well cell culture inserts (transparent PET membrane with 3µm pores; Corning, Flintshire, UK) in 500µl of CCM. In parallel, THP-1 cells were differentiated into a macrophage-like phenotype (dTHP-1) by incubating with 20 mM phorbol 12-myristate-13-acetate (PMA) for 48 h. They were then removed from the flasks using accutase. Before switching to the ALI, 500µl of 1 × 10^5^ cell/ml of dTHP-1 were seeded to the apical side of the insert and allowed to adhere for 2 h before removing all apical medium and implementing an ALI. After switching to the ALI, the co-culture was then incubated at 37°C for 24 h (included in the 48 h recovery phase of the dTHP-1 cells) prior to exposures [41].

### Inflamed model characterisation

Interleukin (IL)-5 (Cat no. 205-IL-005), IL-13 (Cat no. 213-ILB-005) and IL-4 (Cat no. 204-IL-010) from R&D systems (Biotechne, Abingdon, UK) were added at 10ng/ml [42], 5ng/ml [43] and 1ng/ml in the basal medium for 24 h before completing the characterisation of the cultures and determining the optimum concentration to complete the particulate exposures with.

Throughout this manuscript “healthy” is used to differentiate between the unstimulated model and the model stimulated with inflammatory mediators. It is not to suggest that A549 cells are a “healthy” cell. A549 cells were utilised due to their similarities with ATII cells [44] as well as being a well characterised cell line [40].

### Indoor air pollution particulate samples

Reference indoor pollution particles were purchased from the National Institute of Standards and Technology (NIST) (Trace Elements in Indoor Dust—NIST 2583) and all particles were dispersed and sonicated [45]. This standard material was originally collected from vacuum cleaner bags after an indoor dwelling space was vacuumed. Particles were characterised for their heavy metal composition [46] and were determined to be between 473.6 and 1008.23nm depending on dispersant and particulate concentration (Table 1). Particles were dispersed by sonication (Branson Sonifier 250, Ø 13 mm, 400 W output power, 20 kHz) in sterile water. A stock suspension of particles was prepared at a concentration of 2.56mg/mL, which was diluted in sterile water [31] to the desired concentrations.

**Table 1:** Characterisation of NIST SRM 2583 using Dynamic Light Scattering (DLS)

Concentration (µg/ml)	Dispersant	Z-Average (nm) ± SD	Polydispersity Index (PI) ± SD	Peak 1 Mean (nm) ± SD	Peak 2 Mean (nm) ± SD	Size Range (nm)
**2560**	Water	473.60 ± 24.58	0.505 ± 0.010	125.07 ± 42.75	420.37 ± 59.69	68.69–893.0
	0.9% NaCl	606.63 ± 90.43	0.472 ± 0.044	154.00 ± 54.73	501.27 ± 182.12	68.69–768.5
**750**	Water	936.83 ± 314.37	0.550 ± 0.147	250.27 ± 68.72	n/a	169.9–310.7
	0.9% NaCl	562.53 ± 109.20	0.461 ± 0.040	145.77 ± 25.50	449.00 ± 69.00	79.88–893.8
**500**	Water	1008.23 ± 281.98	0.613 ± 0.077	231.33 ± 21.94	n/a	169.9–310.7
	0.9% NaCl	637.73 ± 98.14	0.441 ± 0.058	317.33 ± 56.64	112.75 ± 6.88	50.79–488.7
**250**	Water	600.83 ± 93.37	0.420 ± 0.048	254.07 ± 17.10	n/a	169.9–361.3
	0.9% NaCl	629.80 ± 70.85	0.516 ± 0.136	248.90 ± 23.50	n/a	146.1–361.3

### Dynamic Light Scattering (DLS) analysis

DLS was performed using a ZetaSizer Pro Blue (Malvern Instruments, UK). NIST 2583 was measured at either the stock concentration (2.56mg/ml) or diluted to a concentration of 750, 500 and 250µg/ml (to duplicate the feed concentration applied to the nebuliser). Dilutions were carried out in both distilled water and distilled water spiked with 0.9% NaCl (to give a final concentration of 0.009% NaCl). The DLS Standard Operating Procedure (SOP) was set up according to the instruments operating manual. Then 100µl of the samples were pipetted into micro cuvettes (Sigma-Aldrich, UK) and inserted into the instrument. The instrument was set to equilibrate the sample at 37°C for 2 min prior to measurement initiation and maintained this temperature throughout the measurement process. Each measurement consisted of 10 runs which were averaged, and each sample was performed in triplicate (individually prepared). The instrument attenuator was set to automatic, and analysis of the resulting data was conducted using ZS Xplorer version 3.22 (Malvern, UK).

Exposure concentrations were calculated after consulting the literature for concentrations found within indoor environments [47, 48]. From the literature, these were determined to be between 10-15µg/m^3^, equating to 46.42–60.82mg/cm^2^. The deposition within the alveolar region of the lung [49] was then considered (~ 10% deposition – 4.64–6.08µg/cm^2^) and then further calculations for experimental depositions and limitations within the Cloud12 VitroCell system. Giving the final concentrations of NIST 2583 applied, which were 608, 464, 232ng/cm^2^ (further information in the Supplementary Materials).

**Fig. 2:**
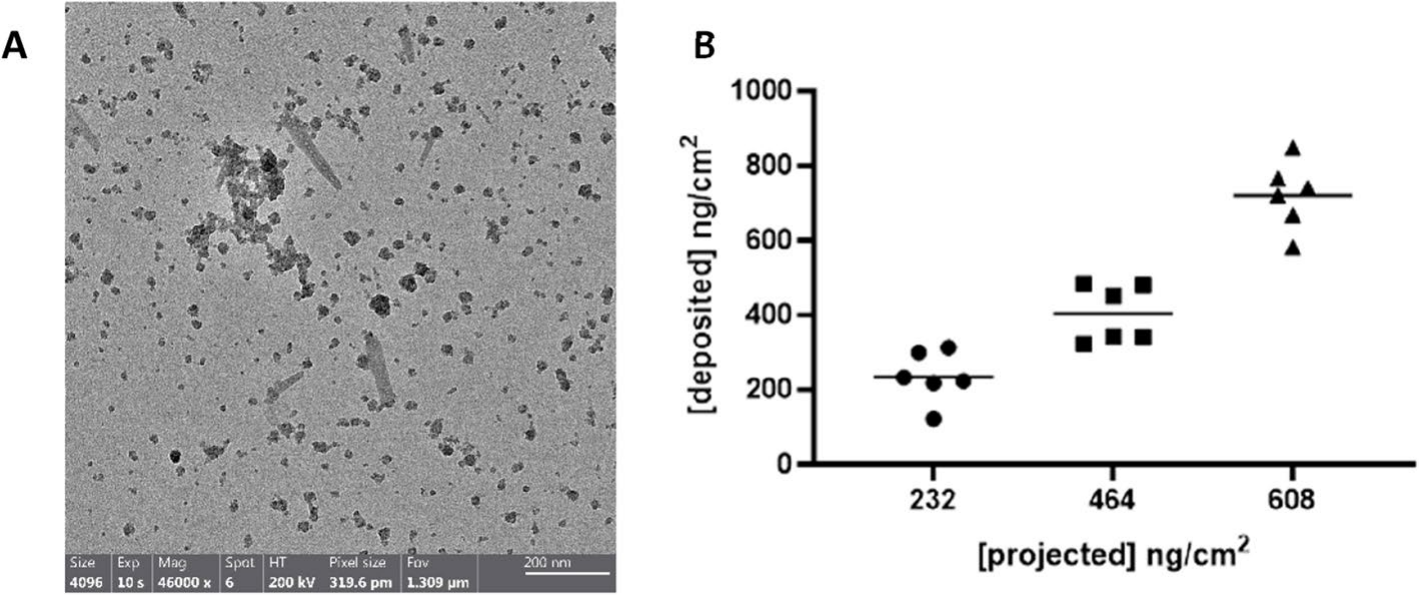
Particulate deposition using the VitroCell Cloud12 System. TEM images of the 608ng/cm.^2^ deposited sample. Scale bar is 200nm (**A**), deposited concentrations and projected deposited values (**B**)

Due to the method of aersolisation and the heterogeneity of the particles (Fig. 2A), the projected concentration was not reached every single exposure and therefore the mean deposited concentration was measured (Fig. 2B) and is what is referred to throughout this manuscript. Various feed concentrations were implemented to achieve the projected concentrations as well as either a single or a double nebulisation of the particles (Table 2), as well as a consistent aersolisation standard operating procedure used [45] for the whole experimental set up.

**Table 2:** Feed concentrations and number of nebulisations required for required deposited concentrations

Aimed for concentrations (ng/cm^2^)	Mean deposited concentrations (ng/cm^2^)	Feed concentration (µg/ml)	Number of nebulisations
232	234.85 ± 68.34	250	1
464	403.93 ± 75.95	750	1
608	720.59 ± 90.11	500	2

#### TEM

To visualise the morphology of the particles deposited by the VitroCell Cloud12 system, TEM grids (carbon-coated copper grids; 300 mesh, CF300-Cu, EMS, USA) were placed into a TEM grid holder (VC5036, VITROCELL® Systems GmbH, Germany) and placed into one of the exposure wells within the Cloud12 VitroCell system. The remainder of the wells were utilised for the in vitro co-culture exposures. Representative TEM images with a resolution of 4096 × 4096 pixels were obtained with an exposure time of 10 s (FEI TALOS F200X TEM operating at 200kV).

### Scanning electron microscopy & energy dispersive X-Ray (EDX) spectroscopy

**Fig. 3:**
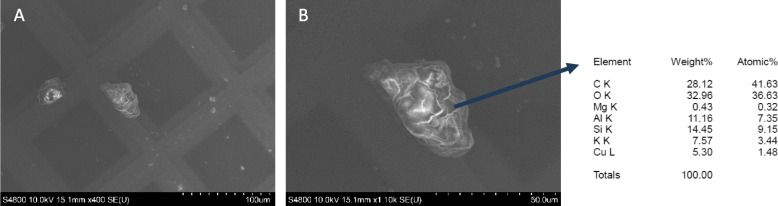
SEM image and EDX analysis of NIST 2583 suspended in distilled water. Scale bar is 100µm (**A**) and 50µm (**B**) with the EDX analysis completed at the site indicated within (**B**)

The samples were prepared (as outlined above in distilled water only) onto carbon-coated copper grids (carbon-coated copper grids; 300 mesh, CF300-Cu, EMS, USA) before being mounted onto an aluminium stub (SEM Clip; 32mm x 10mm x M4 (3 clips)) (Agar Scientific). The sample was then loaded into the SEM vacuum sample chamber. Analysis was performed using the Hitachi Ultra High-Resolution field emission (FE)-SEM model number: S-4800 run at 10kV and 10µA. Image capture was performed in both low magnification and high magnification using the upper SE detector. Energy dispersive X-ray spectroscopy was performed by angling the sample at 20° towards the detector and using the INCA software coupled to the SEM interface (Fig. 3).

### Particle exposures

Cells were exposed through an aerosol exposure (VitroCell Cloud12 system), which entails exposing the cells apically to 200µl of the particle suspension which is then nebulised (using a Aerogen nebuliser head (AG–AL 1000) size rage of 4-6µm) and deposited. Exposures were completed as outlined in Fig. 1. Real-time measurement of deposited particles is acquired via the use of a quartz microbalance (QCM) [50]. For the higher concentration (608ng/cm^2^), repeat exposures were required until the desired deposited concentration was reached within the limitations of the Cloud12 system. Vehicle controls (sterile water spiked with 0.9% NaCl (to give a final concentration of 0.009% NaCl)) were completed—a single exposure for the lower two concentrations (232 and 464ng/cm^2^) and a double exposure for the higher concentration (608ng/cm^2^). Samples were timed for the total length of time for nebulisation to ensure there was a less than 10% deviation from the negative control and no indication of blockage. The aerosol was allowed 6 min to settle, 1 min with the VitroCell Cloud 12 lid removed to allow the QCM to dry and a further 2 min with the lid replaced to allow the measurement to settle. The average of the last 20 seconds of the QCM reading was then taken as the deposited concentration. Incubator controls were included throughout to ensure that the movement between the incubator and the nebulisation were considered within the responses. Assay specific positive controls were also completed throughout, these are highlighted within each assay section below.

Endpoint analyses were completed after 24 h post-exposure at 37°C, 5% CO_2_.

### Biochemical analysis

All samples were processed for viability assessment (Trypan blue exclusion assay), whilst supernatants were collected, centrifuged and the secondary supernatant stored at -80°C for future investigation of specific (pro-)inflammatory mediators.

### Trypan blue exclusion assay

Cellular viability was determined using the trypan blue exclusion assay. Briefly, cells were removed from the cell culture insert using trypsin, centrifuged then resuspended in 1 ml. 10µl of trypan blue dye (0.4%) was added to 10µl of the cell suspension, before being counted with a haemocytometer and percentage viability calculated [40].

### (Pro-)Inflammatory response

The (pro-)inflammatory response of the cells following exposure to NIST 2583 was measured by quantifying the amount of the inflammatory mediators released into the basal medium via Enzyme-Linked Immunosorbent Assay (ELISA). Lipopolysaccharide (LPS) (from *Escherichia coli)* (InVivoGen, Toulouse, France. Cat no. tlrl-3pelps) was used as a positive (pro)-inflammatory control at 1μg/ml on the apical side of the culture (exposed using a quasi-ALI exposure as previously outlined [32, 51]); briefly 21.4µl of LPS was applied apically to the transwell insert. Cell culture supernatant was collected 24 h after exposure and analysed for cytokine levels of IL-8 (Cat no. DY208), IL-6 (Cat no. DY206), IL-10 (Cat no. DY217B), IL-1β (Cat no. DY201), TNF-α (Cat no. DY210), IL-5 (Cat no. DY205), IL-13 (Cat no. DY213), SLPI (Cat no. DY1274-05), and IL-33 (Cat no. DY3625B) using DuoSet kits from R&D systems (Biotechne, Abingdon, UK) according to the manufacturer’s instructions. Samples were analysed in triplicate from three independent experiments (*n* = 3) and absorbance was assessed at 450nm with background correction at 570nm. Previous work within the group has established the lack of interference of Printex90 on ELISA assays, however the supernatant was centrifuged and that supernatant was analysed to further reduce the potential particulate interference. Extrapolation of protein concentration was carried out from a standard curve of known concentrations (IL-8 (0-2000pg/ml), IL-6 (0-200pg/ml), IL-10 (0-2000pg/ml), IL-5 (0-300pg/ml), IL-13 (0-6000pg/ml), SLPI (0-1000pg/ml) and IL-33 (0-1500pg/ml)).

### Barrier integrity

The barrier integrity of each in vitro model was completed using the Blue Dextran assay as previously outlined [32]. Briefly, translocation of blue dextran from the apical to the basal side of the transwell insert was normalised against an empty transwell (with no cells). Blue Dextran (0.5%) (dissolved in PBS) added to the apical surface and CCM to the basal side. Cultures were then incubated for 2 h at 37°C before measuring the absorbance values at 600nm of the basal compartment. This value was then shown as fold over the negative control value. A positive control of 0.05% Ethylenediamine tetraacetic acid (EDTA) was also implemented.

### Immunostaining

Cells were fixed with 4% paraformaldehyde solution (in PBS) at room temperature for 10 min to visualise the cell morphology. Next, the cells were washed with PBS. The cells were subsequently treated with 0.1M glycine in PBS for another 15 min. To permeabilise the cell membrane, the cells were treated with 0.2% Triton X-100 in PBS for 15 min. Phalloidin, Alexa Fluor 633 (A22284; Invitrogen, UK) was used to stain the F-actin cytoskeleton at a 1:200 dilution. DAPI VECTASHIELD (VECTOR Laboratories, USA) was then used to counterstain the nuclei of the cells. The samples were visualized using an inverted laser scanning confocal microscope (LSM 710, Zeiss, Germany).

### In vitro Cytokinesis Blocked Micronucleus (CBMN) assay

Cell survival and cytostasis was assessed alongside micronuclei scoring by relative population doubling (RPD) as described previously [52]. This ensured that the cytostasis of the cell line remained within the recommendation of OECD test guideline 487 of 55 ± 5%. The CBMN assay was completed as previously described [53] 24 h post exposure to the particles (*ca.* 1-cell cycle). Mitomycin-C (MMC) at 0.01μg/ml was used as a positive control. After exposure, cells were washed in 1 × phosphate buffered saline (PBS) three times and re-suspended in fresh media containing 3μg/ml cytochalasin B for a further 24 h. The cells were then trypsinised, pelleted by centrifugation (1200xg for 5 min) and washed twice in PBS. They were then fixed in 3% paraformaldehyde and permeabilized with Triton X100. Cells were washed with PBS prior to staining with 1µg/ml (1:100 dilution) of anti-human CD324 (e-Cadherin) with a conjugated FITC fluorophore (BioLegend®, San Francisco, USA). Following washing and resuspension in 1mL of PBS, cells were pipetted on to slides and coverslips were attached with DAPI VECTASHIELD (VECTOR Laboratories, USA). Cell imaging and micronuclei identification was undertaken using an Axioimager Z2 fluorescent microscope with a one megapixel charged coupled device camera (Carl Zeiss, UK). Slides were prepared and scored for the presence of micronuclei in binucleated cells using the automated Metafer image analysis system (Metasystems, Carl Zeiss Ltd) as described previously by [54]. All experiments were performed in triplicate (*n* = 3) with 1000 binucleuate cells scored per replicate (3000 binucleate cells in total) for each treatment.

### Comet assay

The comet assay was completed as outlined previously [55]. Briefly, microscope slides were pre-coated with agarose (0.5% Normal Melting Point Agarose (99.5ml Water + 0.5g NMP-Agarose) before being left overnight at room temperature. At 24 h post particulate exposure, cells were embedded on the pre-coated slide with 0.8% low melting point agarose before being incubated at 4°C for an hour in lysis solution. Slides were then placed in the alkaline solution and electrophoresis was run (20 min at 1V/cm). The slides were then washed (cold PBS, cold dH_2_O (5 min), then left at room temperature horizontally overnight), fixed and stained with SYBRGold (0.1ul/ml in TE Buffer (Tris–EDTA) = 2.5mM Tris, 4mM Na2EDTA) (ThermoFisher, UK) before scoring 50 cells per gel dot. The %DNA in the tail was subsequently analysed using Comet Assay IV (Perspective Instruments Ltd, version 4.3.2).

### Statistical analysis

All data are presented as the mean ± standard deviation (SD) unless stated otherwise. All endpoints were assessed following three independent cell cultures (*n* = 3). Statistical analyses were performed using GraphPad Prism 10 (GraphPad Software Inc., La Jolla, USA) software. A one-way analysis of variance (ANOVA) with subsequent Tukey’s multiple comparisons *post-hoc* test was performed for each endpoint. Results were considered significant if *p* < 0.05.

## Results

### Unstimulated model exposure

#### Viability and membrane integrity

**Fig. 4:**
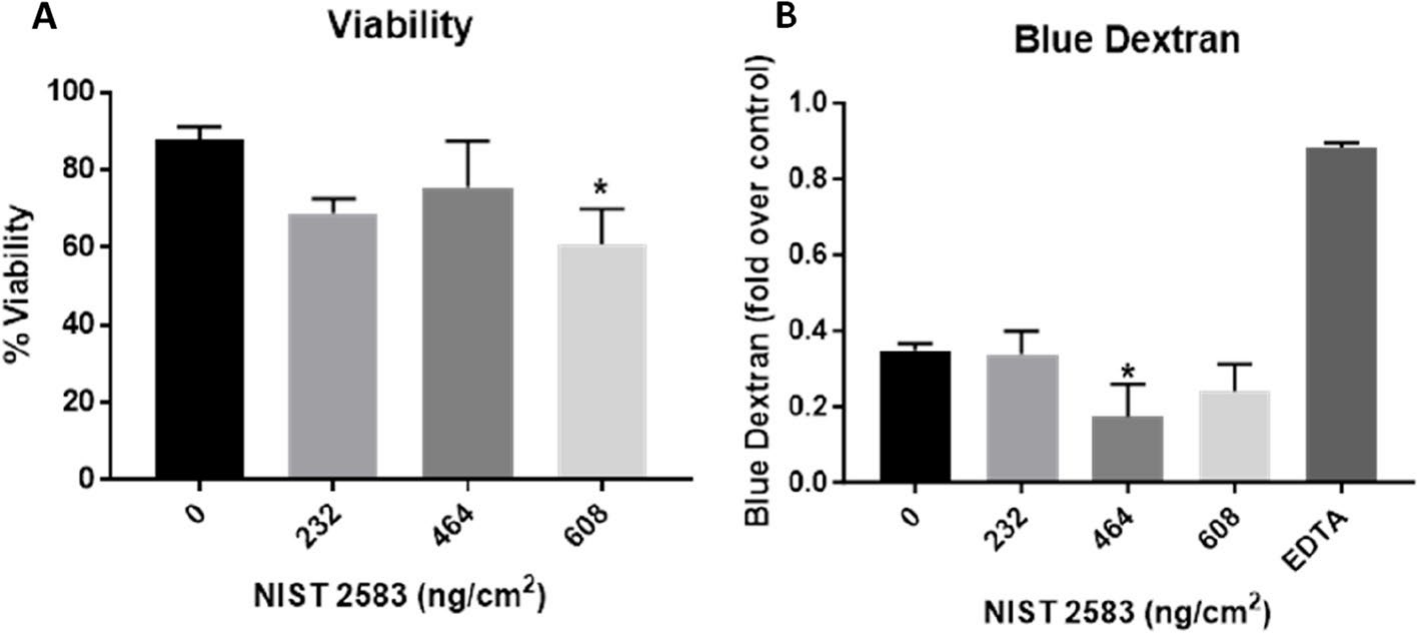
Viability and membrane integrity, 24 h post exposure of NIST 2583 on the unstimulated A549 + dTHP-1 co-culture. Cells were exposed for 24 h at an ALI using the VitroCell Cloud12, before analysing cytotoxicity (**A**) and membrane integrity (blue dextran) (**B**). *n* = 3 with all assays performed in triplicate. The data is presented as the mean ± standard deviation. Significance is denoted as the following: compared to the medium control *p* < 0.01(*)

**Fig. 5:**
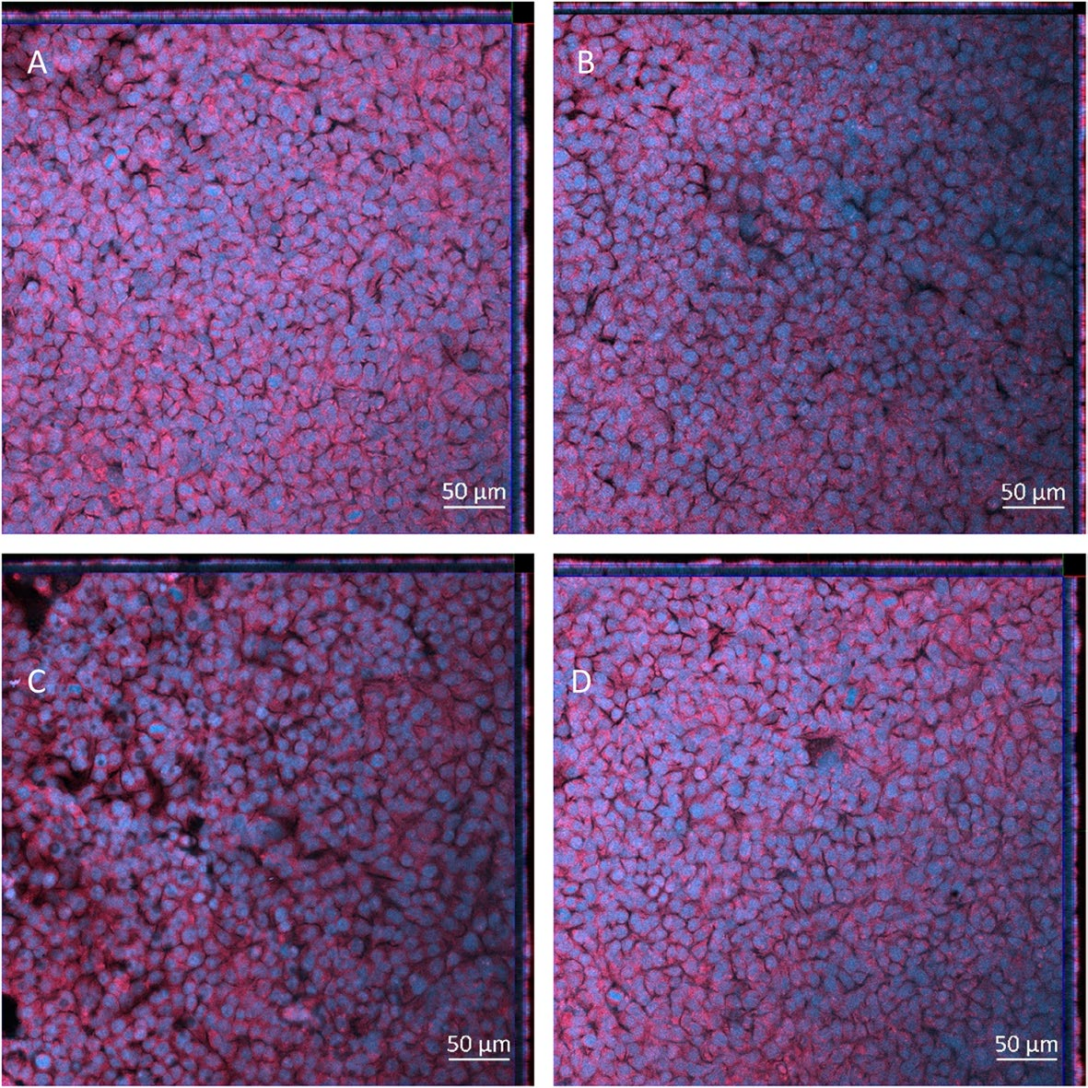
Cell morphology 24 h post exposure of NIST 2583 on the unstimulated A549 + dTHP-1 co-culture. Cells were exposed for 24 h at an ALI using the VitroCell Cloud12—Negative control (**A**), 232ng/cm^2^ (**B**), 464ng/cm^2^ (**C**), and 608ng/cm^2^ (**D**). DAPI is indicated by blue while phalloidin is indicated by red. Scale bar is 50µm

There was a significant decrease (*p* < 0.01) in the cellular viability after exposure to the highest concentration of NIST SRM 2583 (608ng/cm^2^) when compared to the negative control. There were also slight decreases in the viability at the lower two concentrations of NIST 2583 (232 and 464ng/cm^2^), however these were not significant (*p* > 0.05) (Fig. 4A). The higher two concentrations (464 and 608ng/cm^2^) caused an increase in the membrane integrity; however, this was only significantly increased (*p* < 0.01) after exposure to 464ng/cm^2^ when compared to the negative control (Fig. 4B and Figure S1). Changes to the membrane integrity can also be identified within Fig. 5C, compared to the control and the other exposures (Fig. 5A-D).

### Genotoxicology

**Fig. 6:**
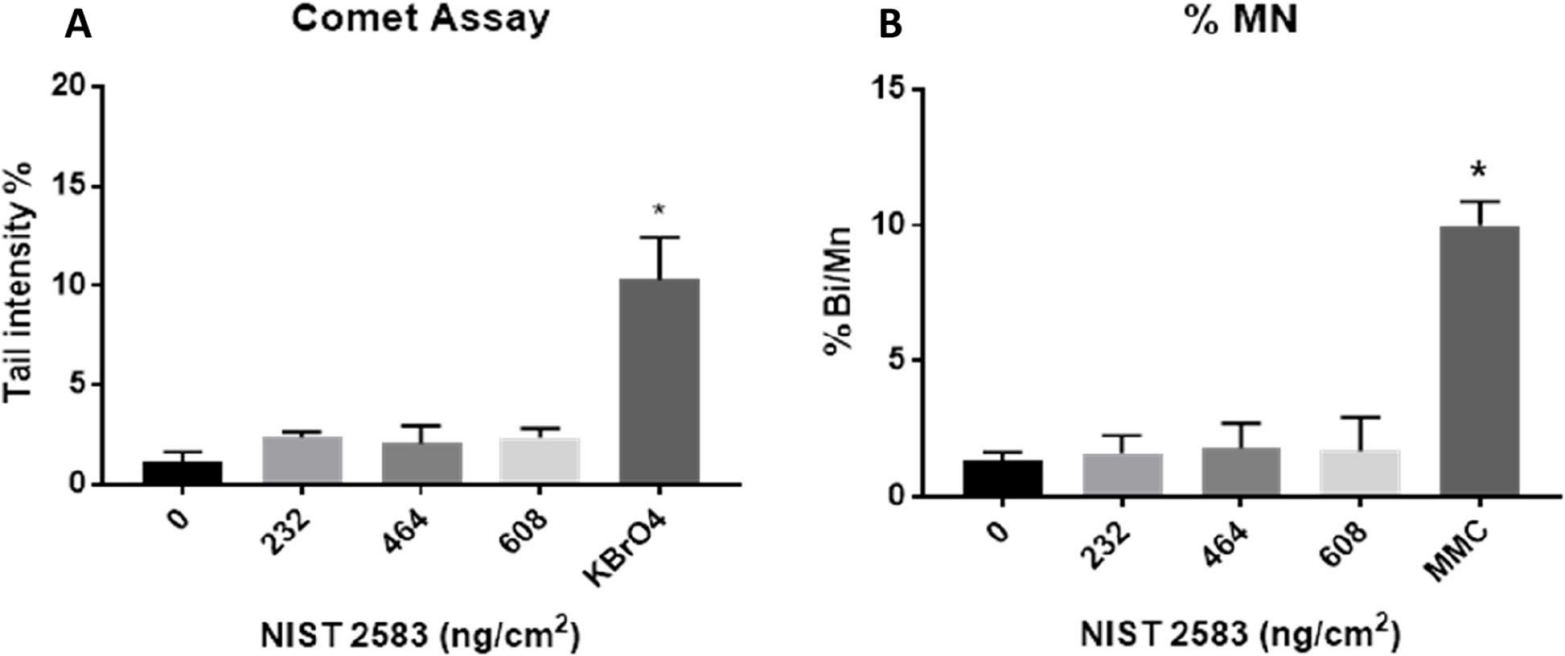
Genotoxicology assessment 24 h post exposure of NIST 2583 on the unstimulated A549 + dTHP-1 co-culture. Cells were exposed for 24 h at an ALI using the VitroCell Cloud12, before completing the comet assay (**A**) and CBMN assay (**B**). *n* = 3 with all assays performed in triplicate. The data is presented as the mean ± standard deviation. Significance is denoted as the following: compared to the negative control *p* < 0.01(*)

Both repairable double strand DNA breaks (comet assay) and non-repairable double strand DNA breaks (Mn assay) were investigated (Fig. 6A and B respectively). There were no significant impacts (*p* > 0.01) upon cellular DNA when using either assay after exposure to any of the tested concentrations of NIST 2583 when compared to the negative control. There were, however, significant responses in the appropriate positive controls for both the Comet and the Mn assay that were implemented for each of the assays.

### (Pro-)inflammatory responses

**Fig. 7:**
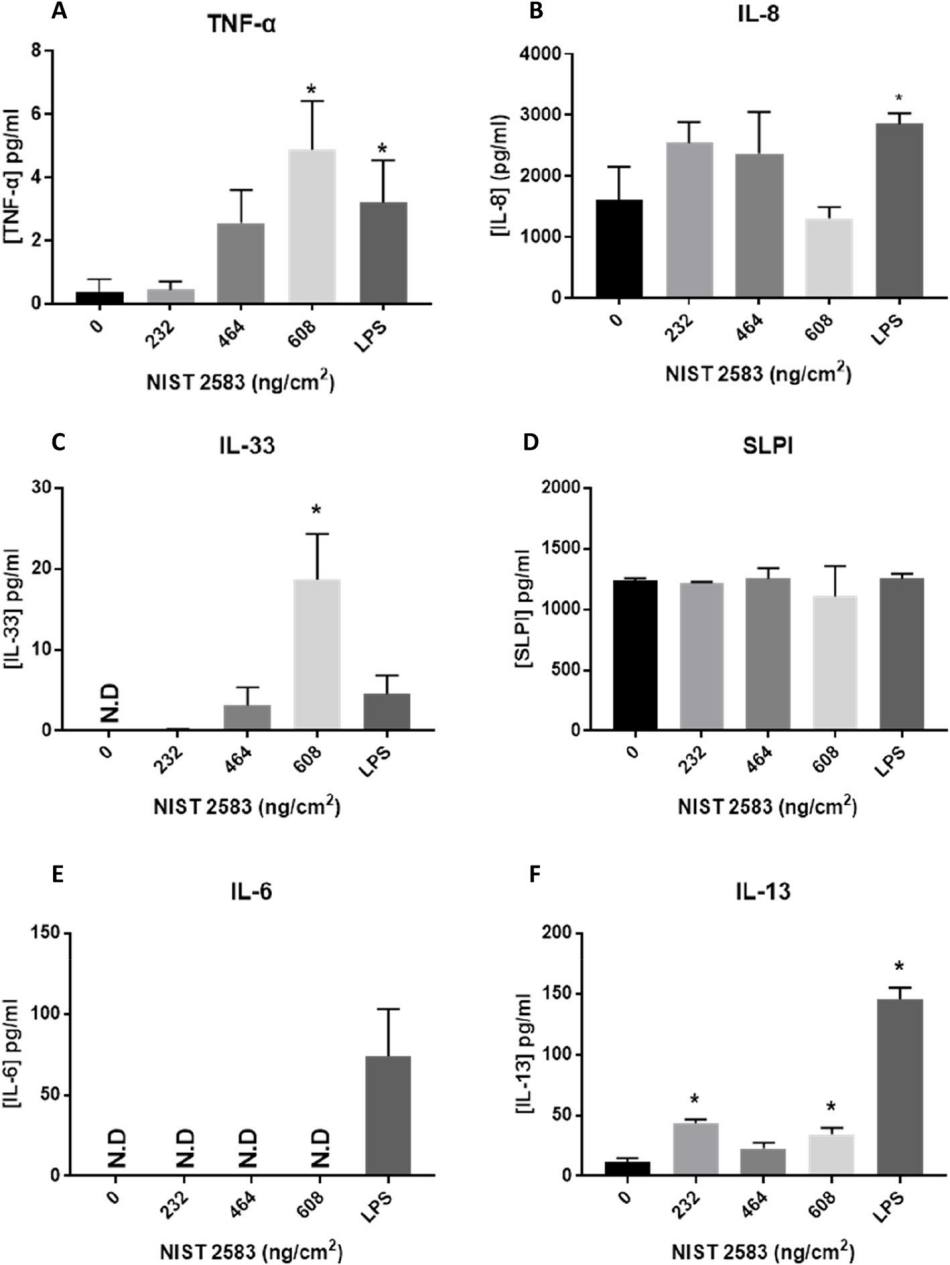
Pro-inflammatory mediators of interest 24 h post exposure of NIST 2583 on the unstimulated A549 + dTHP-1 co-culture. TNF-α (**A**), IL-8 (**B**), IL-33 (**C**), SLPI (**D**), IL-6 (**E**) and IL-13 (**F**) basal concentration 24 h post exposure after the particle exposure (onto the apical side). Cells were exposed for 24 h at an ALI using the VitroCell Cloud12 and left to incubate for 24 h. *n* = 3 with all assays performed in triplicate. The data is presented as the mean ± standard deviation. Significance is denoted as the following: compared to the medium control *p* < 0.01(*). N.D – non-detection

Concentrations of various inflammatory mediators and alarmins were measured in the cell supernatant post exposure. The concentration of IL-6 was below detection limits after exposure to all concentrations of NIST 2583 (Fig. 7E). There was a significant increase (*p* < 0.01) in IL-13 after exposure to 232 and 608ng/cm^2^ (Fig. 7F) when compared to the negative control There was a significant increase (*p* < 0.01) in both IL-33 and TNF-α after exposure to 608ng/cm^2^ (Fig. 7C and A respectively). There was no change in the SLPI concentration after any of the exposures (Fig. 7D). There were no significant changes in the IL-8 concentration after exposures, but there was an increase at the two lower concentrations (232 and 464ng/cm^2^) and no change after 608ng/cm^2^ (Fig. 7B).

### “Inflamed” (Stimulated) model characterisation

#### Viability and membrane integrity

**Fig. 8:**
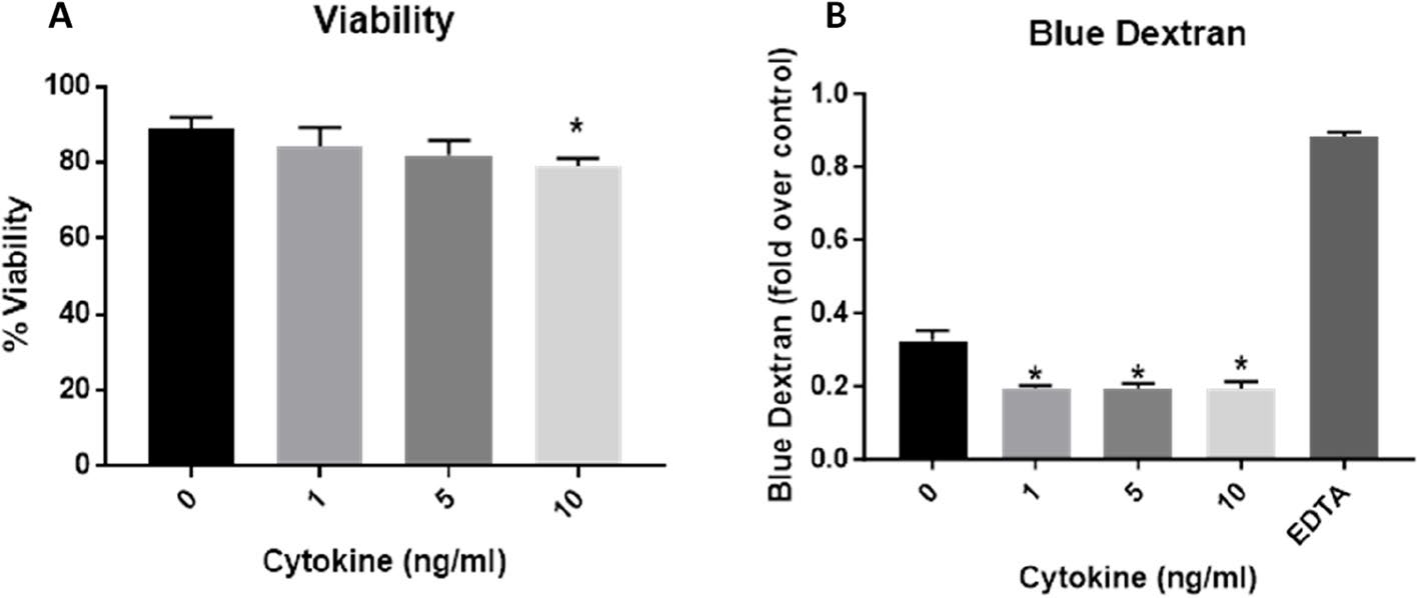
Viability and membrane integrity, 24 h post exposure of 1, 5, and 10ng/ml of IL-5, IL-13 and IL-4 on the apical side of the stimulated A549 + dTHP-1 co-culture. Cells were exposed for 24 h at an ALI using before analysing cytotoxicity (**A**) and membrane integrity (blue dextran) (**B**). *n* = 3 with all assays performed in triplicate. The data is presented as the mean ± standard deviation. Significance is denoted as the following: compared to the medium control *p* < 0.01(*)

The addition of IL-5, IL-13 and IL-4 onto the apical side of the A549_dTHP-1 was assessed before exposure of the model to the NIST 2583 particles. After 24 h post-exposure to the mediators, a significant (*p* < 0.01) decrease in the viability of the co-culture after incubation with 10ng/ml was noted, when compared to the negative control. This was however still ~ 80% viable (Fig. 8A). There was however a significant increase (*p* < 0.01) in the membrane integrity after incubation of all concentrations of the mediators when compared to the negative control, but this was not a dose dependant increase (Figs. 8B, S2 and 9A-D).

**Fig. 9:**
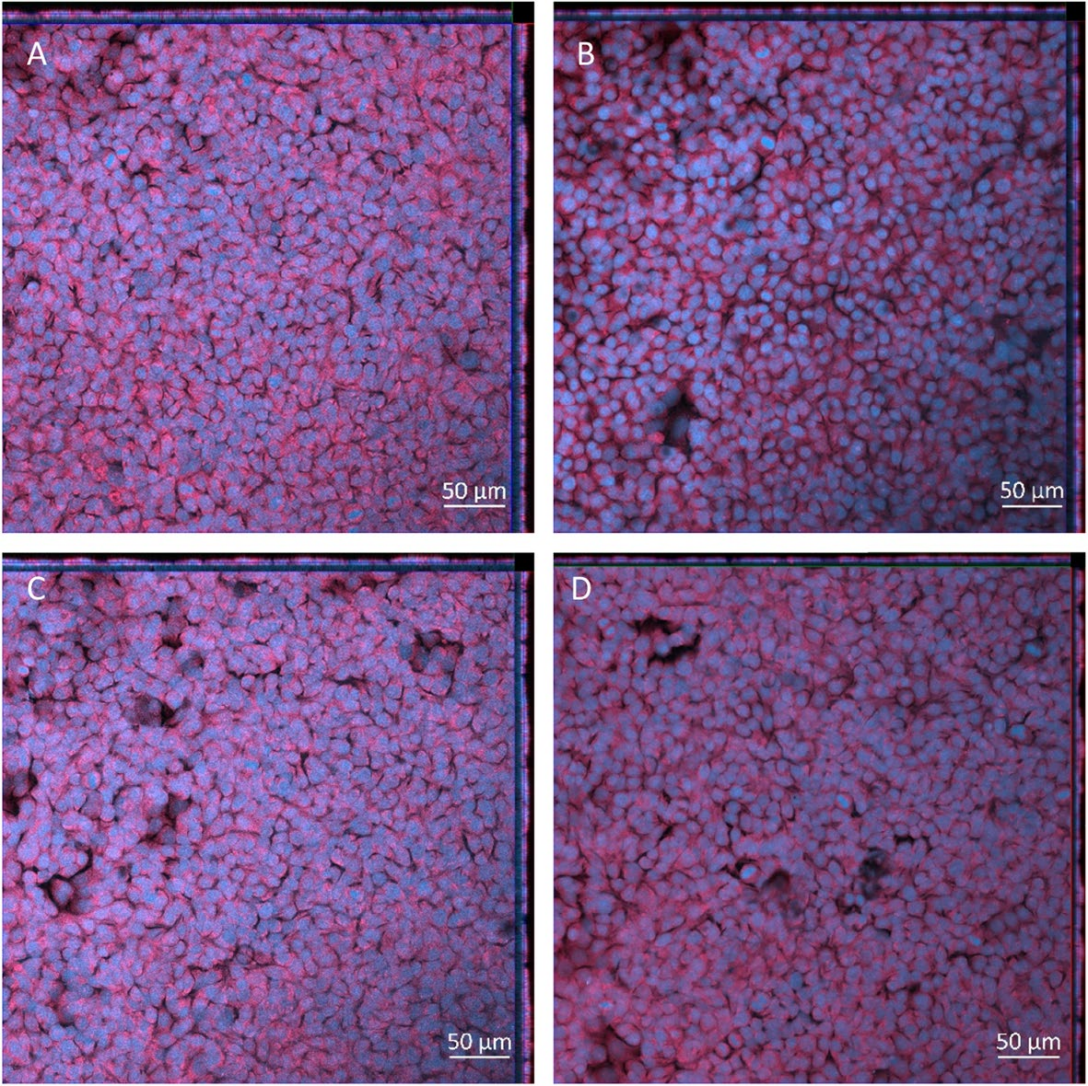
Cell morphology 24 h post exposure of 1, 5, and 10ng/ml of IL-5, IL-13 and IL-4 on the apical side of the stimulated A549 + dTHP-1 co-culture—Negative control (**A**), 1µg/ml (**B**), 5µg/ml (**C**), and 10µg/ml (**D**). DAPI is indicated by blue while phalloidin is indicated by red. Scale bar is 50µm

#### (Pro-)inflammatory responses

**Fig. 10:**
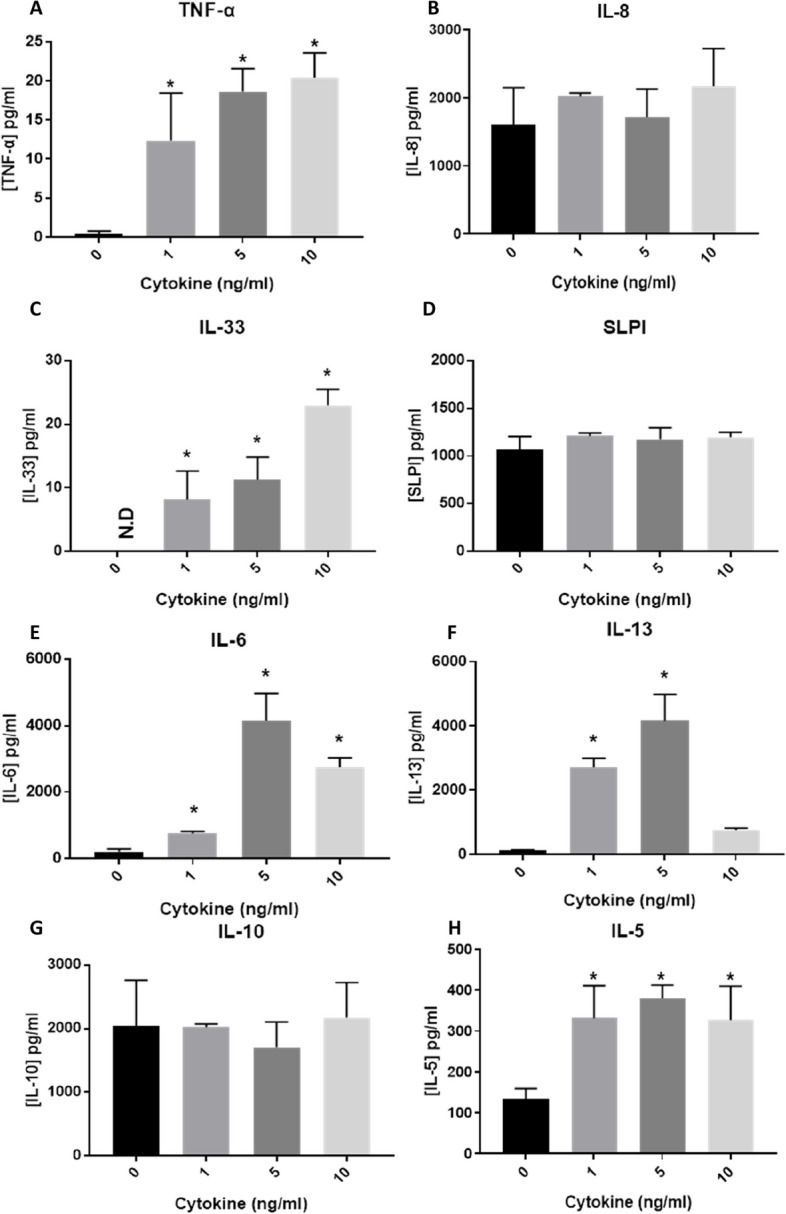
Inflammatory mediators of interest 24 h post exposure to 1, 5, and 10ng/ml of IL-5, IL-13 and IL-4 (onto the apical side). TNF-α (**A**), IL-8 (**B**), IL-33 (**C**), SLPI (**D**), IL-6 (**E**), IL-13 (**F**), IL-10 (**G**) and IL-5 (**H**) basal concentration 24 h post exposure. *n* = 3 with all assays performed in triplicate. The data is presented as the mean ± standard deviation. Significance is denoted as the following: compared to the medium control *p* < 0.01(*)

Concentrations of various inflammatory mediators and alarmins were measured in the cell supernatant 24 h after stimulation with the mediators. There was a significant dose-dependent increase (*p* < 0.01) in the concentration of IL-33 and TNF-α (Fig. 10C and A respectively) when compared to the medium control. A significant increase (*p* < 0.01) in the concentration of IL-6 (Fig. 10E), and IL-13 (Fig. 10F) after both 1 and 5ng/ml of the mediators compared to the negative control and in a dose dependant manner. There was a significant (*p* < 0.01) increase in IL-6 compared to the negative control after 10ng/ml, but this exposure elicited no significant increase of IL-13 (Fig. 10E and F respectively). There were no changes in the concentrations of SLPI (Fig. 10D), IL-8 (Fig. 10B) or IL-10 (Fig. 10G) at any of the concentrations of mediators when compared to the negative control or the other concentrations. All concentrations of the mediators induced a significant increase (*p* < 0.01) in IL-5 compared to the negative control (Fig. 10H). Taken together, this indicated that 5ng/ml induced the most consistent (pro-)inflammatory responses without a significant decrease in viability and therefore was implemented in future “inflamed” model exposures.

### “Inflamed” model exposure

#### Viability and membrane integrity

**Fig. 11:**
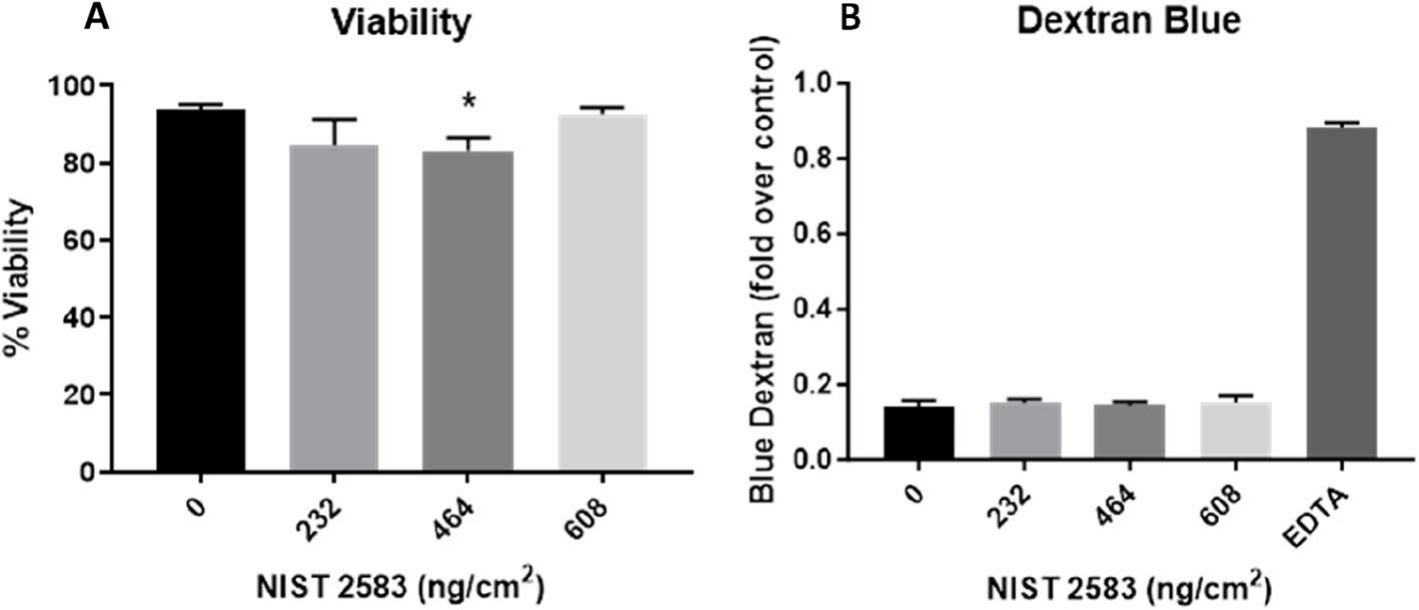
Viability and membrane integrity, 24 h post exposure of NIST 2583 on an “inflamed” A549 + dTHP-1 co-culture. Cells were exposed for 24 h at an ALI using the VitroCell Cloud12, before analysing cytotoxicity (**A**) and membrane integrity (blue dextran) (**B**). *n* = 3 with all assays performed in triplicate. The data is presented as the mean ± standard deviation. Significance is denoted as the following: compared to the medium control *p* < 0.01(*)

After all exposures to NIST 2583 there was a decrease in the viability of the co-culture (which did not fall below ~ 80%), this was significant (*p* < 0.01) 24 h post exposure to 464ng/cm^2^ compared to the negative control only (Fig. 11A). There were no changes in the membrane integrity after any of the NIST 2583 exposures when compared to the negative control (Figs. 11B, S3 and 12A-D).

**Fig. 12:**
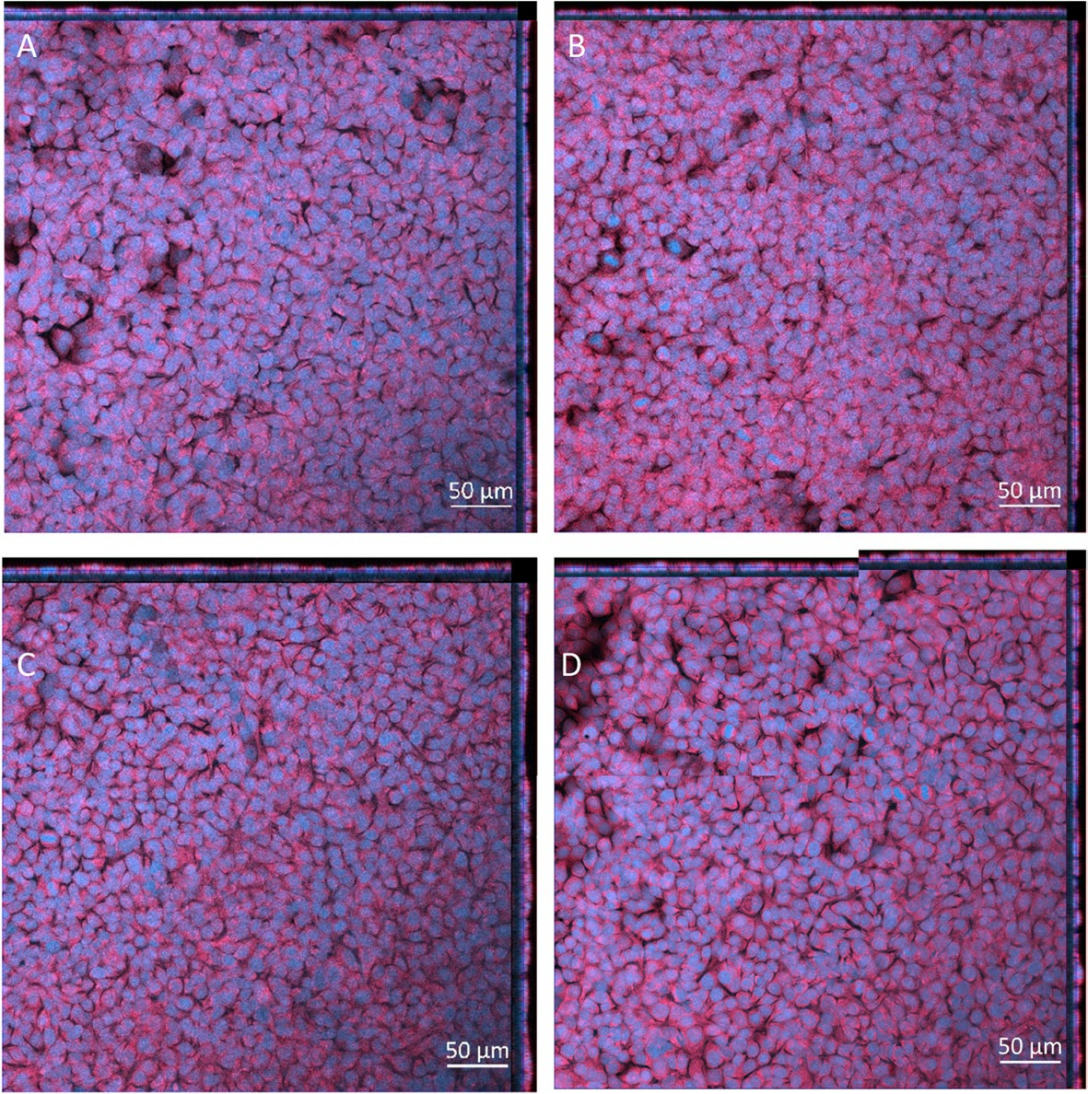
Cell morphology 24 h post exposure of NIST 2583 on an “inflamed” A549 + dTHP-1 co-culture. Cells were exposed for 24 h at an ALI using the VitroCell Cloud12—Negative control (**A**), 232ng/cm^2^ (**B**), 464ng/cm^2^ (**C**), and 608ng/cm^2^ (**D**). DAPI is indicated by blue while phalloidin is indicated by red. Scale bar is 50µm

#### Genotoxicology

**Fig. 13:**
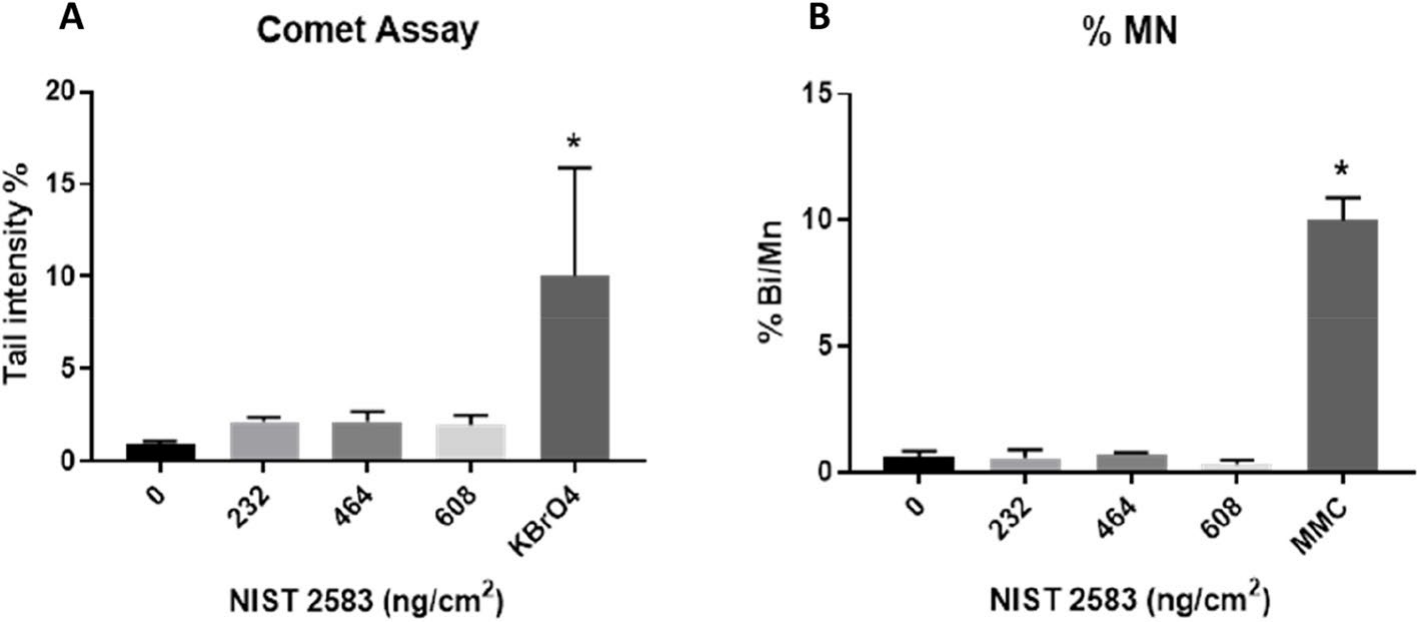
Genotoxicology assessment 24 h post exposure of NIST 2583 on an “inflamed” A549 + dTHP-1 co-culture. Cells were exposed for 24 h at an ALI using the VitroCell Cloud12, before completing the comet assay (**A**) and CBMN assay (**B**). *n* = 3 with all assays performed in triplicate. The data is presented as the mean ± standard deviation. Significance is denoted as the following: compared to the negative control *p* < 0.01(*)

There were no changes in either assay after exposure to any of the concentrations of NIST 2583 when compared to the negative control for either assay (Fig. 13A and B).

#### (Pro-)inflammatory responses

**Fig. 14:**
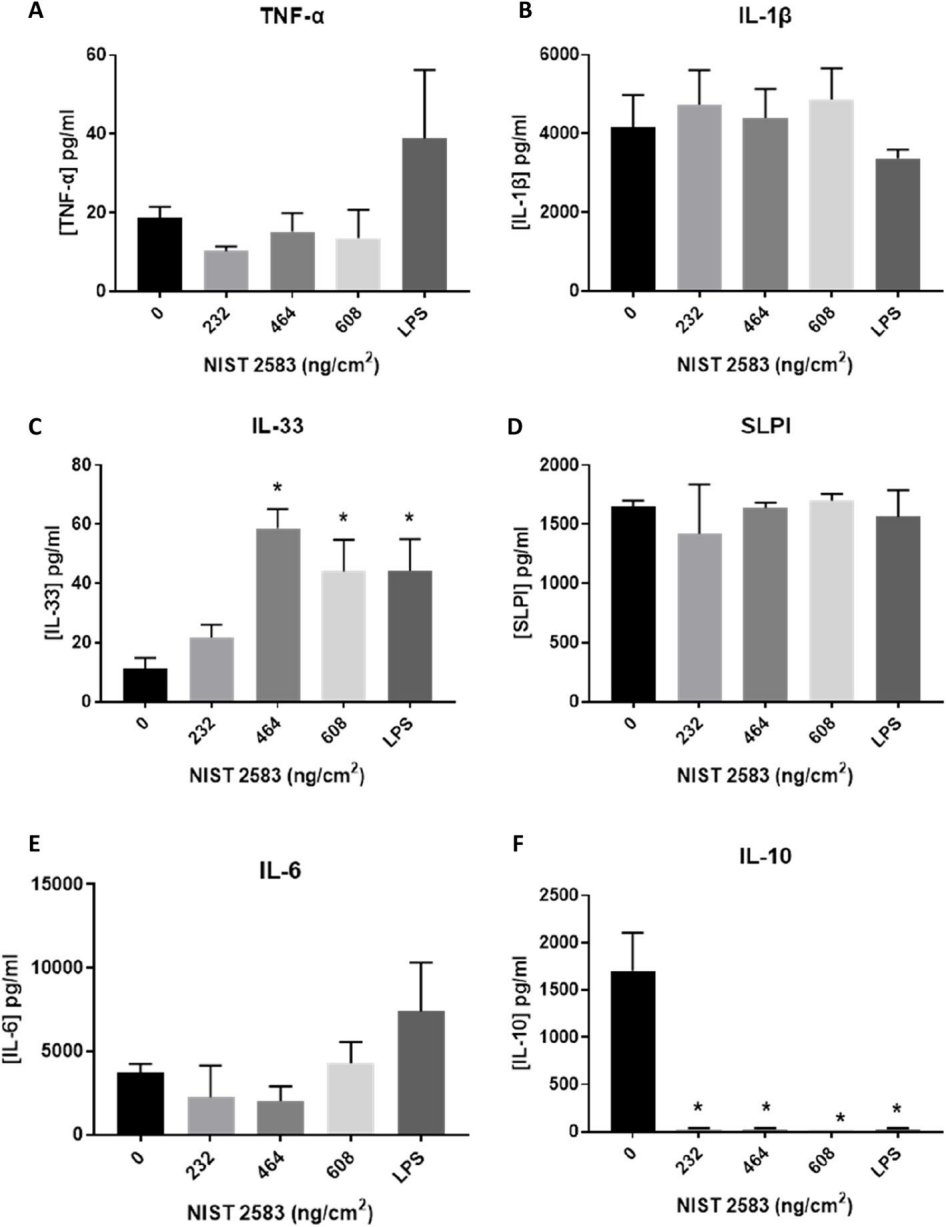
Inflammatory mediators of interest 24 h post exposure of NIST 2583 on an “inflamed” A549 + dTHP-1 co-culture. TNF-α (**A**), IL-1β (**B**), IL-33 (**C**), SLPI (**D**), IL-6 (**E**) and IL-10 (**F**) basal concentration was analysed 24 h post particle exposure (onto the apical side). Cells were exposed for 24 h at an ALI using the VitroCell Cloud12 and left to incubate for 24 h. *n* = 3 with all assays performed in triplicate. The data is presented as the mean ± standard deviation. Significance is denoted as the following: compared to the medium control p < 0.01(*)

Concentrations of various inflammatory mediators and alarmins were measured in the cell supernatant 24 h after stimulation with the mediators and exposure to 232, 464 and 608ng/cm^2^ NIST 2583. There were no significant changes in the concentration of IL-6 (Fig. 14E), IL-1β (Fig. 14B), SLPI (Fig. 14D), or TNF-α (Fig. 14A). There was a significant increase in IL-33 supernatant concentration (*p* < 0.01) after exposure to 464 and 608ng/cm^2^ when compared to the negative control (Fig. 14C). There was a significant decrease (*p* < 0.01) in the IL-10 concentration after exposure to all NIST 2358 concentrations when compared to the negative control (Fig. 14F).

## Discussion

The initial aim of this study was to determine the potential toxicological effect of a standard indoor air pollution particle (NIST 2583) on a previously characterised co-culture model of the alveolar region of the lung (A549_dTHP-1 model), using a physiologically relevant exposure method and concentration. In addition, this study aimed to characterise and implement an “inflamed” model of the alveolar region and determine if the toxicological effects of NIST 2583 remained consistent or if this inflamed state influenced the biological endpoints. After completing both the characterisation and the exposure of the “inflamed” model, there was indication that the biological endpoints post exposure to this standard particle where influenced by the model’s phenotype (Table 3).

**Table 3:** Summary of study findings indicating decreases (↓), increases (↑), and no changes ( ↔)Significance is indicated by appropriate symbol (* compared to negative control). ND is non-detectable, and n/a is not applicable

	Non-stimulated A549_dTHP-1 model			“inflamed” A549_dTHP-1 model		
	232ng/cm^2^	464ng/cm^2^	608ng/cm^2^	232ng/cm^2^	464ng/cm^2^	608ng/cm^2^
Viability	↓	↓	↓*	↓	↓*	↔
Membrane integrity	↔	↑*	↑	↔	↔	↔
Mn Assay	↔	↔	↔	↔	↔	↔
Comet Assay	↔	↔	↔	↔	↔	↔
IL-6	ND	ND	ND	↔	↓	↔
IL-1β	n/a	n/a	n/a	↔	↔	↔
TNF-α	↔	↑	↑*	↓	↔	↔
IL-33	↔	↑	↑*	↑	↑*	↑*
SLPI	↔	↔	↔	↔	↔	↔
IL-8	↑	↑	↔	n/a	n/a	n/a
IL-10	n/a	n/a	n/a	↓*	↓*	↓*
IL-13	↑*	↑	↑*	n/a	n/a	n/a

### Indoor air pollution exposure

There is a known link between indoor air pollution and poor health outcomes. A recent study has determined a link between increases in PM_2.5_ and child absences in a school setting (for every 1µg/m^3^ of PM_2.5_ in the classroom there was an increase of 7.37 days of absence) [56]. Indoor PM has also been linked to various outcomes, including premature death in cardiovascular and pulmonary patients, as well as exacerbations of diseases linked to these organs (*e.g.,* increases in heart attacks and asthma attacks) [8]. NIST SRM 2583 is a reference material used in the herein study to assess the potential impacts indoor air pollution particles may have. Currently there limited work within the literature investigating the biological or toxicological effects of these particles either in vivo or in vitro*.* However, there has been work completed on collected particles from various sources.

These various sources have varying biological and toxicology effects depending on their composition and how they are produced. The use of a reference material also comes with its own problems; however, it is important to utilise a material that will remain consistent across different models and exposure scenarios. It may not be completely reflective of a modern living environment, but components of this reference material will not change (such as the presence of endotoxin for example) across living environments. It also allows a baseline potential of toxicity of indoor compared to outdoor particulate matter to be established. A study using a monoculture A549 model with a submerged exposure (an exposure that is not physiologically relevant), determined that PM_10_ collected from burning indoors induced cell death at lower concentrations than the outdoor burning PM_10_ collected [57]. Using the same cell types (A549 cells), and collected indoor PM_10_ without burning identified no significant change in the viability of the cells, but there was a dose depend decrease in the viability [58]. Another study implementing BEAS2B cells and also using a submerged exposure to occupational PM produced via shredding paper determined that there was no change in cellular viability when exposed to this PM [59]. From our study (Fig. 4A), we determined that there was a decrease in viability of the co-culture model, but this was only significant at the highest concentration. It is important to note that the study completed here was done under physiologically relevant conditions (air–liquid interface) and using a physiological exposure method (aerosol), whereas the previously mentioned studies were all monocultures and exposed under submerged conditions.

As well as PM being a component there are various other elements of air pollution that could be considered. Ozone is one such element. Using A549 cells there was a significant increase in micronuclei frequency after exposure to silica PM_2.5_ and PM_2.5_ + O_3_. This was not seen with the comet assay, and the exposure to PM_2.5_ + O_3_ decreased the genotoxicity compared to the PM_2.5_ alone. However, this was in a submerged culture and not at an air–liquid interface [60]. This suggests that the combination of PM_2.5_ + O_3_ had the potential to induce more non-repairable double strand breaks than the PM_2.5_ alone. A study conducted comparing mice exposed via intratracheal instillation saw no genotoxic effects of either indoor or outdoor air pollution particles that were collected via filters [61]. From our study of PM alone, we identified no changes in either the comet assay or the Mn assay (Figs. 6A and B and 13A and B) indicating no genotoxic of effect of these particles alone. However, previous studies indicate the potential for genotoxic effects to be identified if these particles we co-exposed with other indoor air pollutants [60]. There may also be the potential for the environment in where the particles are collected to influence the biological outcome once again. Using human peripheral lymphocytes, there was a link identified between the potential genotoxicity of collected indoor PM_2.5_ from various occupational settings and the heavy metal content (with Zn and Pb concentrations indicating the genotoxic potential of the collected particles) [62].

IL-33 is known to promote a type II response, leading to increase IL-4, IL-13 and IL-9 production [63]. IL-33 is known to be linked to necrosis and necroptosis is a mechanism of release [64]. It is produced via secretion or via cellular death [65]. At the highest concentration of NIST 2583 alone in this study there was a significant increase in cell death (Fig. 4A), which was coupled with a significant increase in IL-33 (Fig. 7C), IL-13 (Fig. 7F) and TNF-α (Fig. 7A), indicating that the highest concentration of NIST 2583 (608µg/cm^2^) is inducing a type II response in this model. TNF-α is secreted by activated macrophages and is implemented in the exacerbation of various lung diseases (such as bronchitis, COPD and asthma) [66]. TNF-α can induce the expression of IL-33 mRNA in the skin [67], as well as IL-33 can promote the production of TNF-α by macrophages [68]. The concentration of baseline TNF-α and IL-33 (Fig. 14A and C respectively) in the “inflamed” model is either higher than or the same as the highest concentration of NIST exposure in the non-stimulated model (Fig. 7A and C respectively) ~ 5pg/ml compared to ~ 20pg/ml and ~ 20pg/ml respectively. This means that any dose dependant responses in TNF-α in the “inflamed” model has the potential to be masked by this high baseline concentration. Whereas there is still a dose dependant response of IL-33 after exposures to NIST 2583 (Fig. 14C) which is significant at the higher two concentrations (464 and 608ng/cm^2^) compared to the negative control. This further highlights the influence of IL-33 in this model and response to these indoor air pollution particles and the induction and enhancement of a type II response due to exposure of these NIST 2583 particles. This type II response is then induced by a lower concentration in the “inflamed” model when compared to the healthy model.

IL-33 and IL-13 are known to synergistically enhance the production of IL-33 in bronchial epithelial cells, indicating feed-back loops that can continuously induce lung inflammation [69]. ST2 is the receptor for IL-33 and can be found on various cell types, including macrophages and epithelial cells and can help regulate both the innate and adaptive immune system [70]. Therefore, once these mediators have been induced (which they are in both the “inflamed” and normal model – Figs. 7C and F and 14 C respectively) there are multiple feedback loops which lead to the continuous production of these mediators.

IL-10 is an anti-inflammatory cytokine, its role is related to the reducing the inflammatory response to a pathogen or stimulation [71]. Therefore, if there is a decrease in the concentration of IL-10 (Fig. 14F) after NIST 2583 exposure when compared to the mediator stimulated cells, it is possible that the exposure to these specific particles is reducing the concentration of anti-inflammatory cytokines and actually contributing to the reducing of anti-inflammatory mediators within the system. Air pollution exposure in children has been linked to the methylation of the genes associated with IL-10 [72], and indoor exposure to polycyclic aromatic hydrocarbons (PAHs) lead to a significant decrease in mRNA of IL-10 [73].

In a study investigating the effects of chronic air pollution exposure in an older population (female, 69–79 years), it was identified that there was a significant increase in leukotriene (LT)B_4_ associates with particulate matter exposure [74]. LTB_4_ is a known chemoattractant for neutrophils and macrophages and is important in acute inflammatory responses [75]. This indicates the potential activation of macrophages and therefore the release of macrophage mediators (as seen in Fig. 7A).

### Characterisation and implementation of the “inflamed model”

There are numerous inflammatory diseases that are difficult to mimic in vitro and therefore models are beginning to be developed to represent human inflammatory diseases in vitro*.* Some of these implement the use of inflammatory mediators to stimulate the model or functionally change the model, for example; skin models aiming to focus on atopic dermatitis and psoriasis [76]; using mediators and immune cells during mesenchymal stem cell differentiation to mimic an inflammatory osteoarthritis joint [77, 78]; investigating an inflamed blood brain barrier [79]; and respiratory tract infections [80]. However, there are very few (if any) publications that stimulate alveolar models with inflammatory mediators to develop a model with an inflammatory phenotype. The majority of in vitro models looking at human airway diseases and inflammation focus on the immune cells that are activated within these states [81, 82], not the background increased inflammatory mediators that are found within these conditions. Type II inflammatory mediators (as used within this study – IL-4, IL-5 and IL-13) are known to exacerbate asthma and inhibition of these pathways can reduce asthma exacerbations seen clinically [83]. They have also been implemented in type-II diseases such as IgE production and eosinophilia [84]. There was a significant increase in the mediators associated with these diseases after type II cytokine incubation, such as IL-33 (Fig. 10C), and TNF-α (Fig. 10A), with no changes in anti-inflammatory cytokines such as IL-10 (Fig. 10G). This indicates that the model used within this study has a “inflamed” phenotype and aims to mimic the responses in the forementioned airway diseases.

## Conclusion

The exposure to NIST 2583 in a non-stimulated model of the alveolar region induced biological responses that are known to also be induced in human studies by inhalation of air pollution particulates. The stimulation of this model lead to an increased upregulation of specific inflammation markers (such as IL-33) and indicated the exposure to NIST 2583 had additional influences on this response compared to the stimulated model alone. This taken together indicates the potential for indoor air pollution particles on a non-stimulated model to induce an inflammatory response similar to that seen within an inflamed human airway, with an inflamed airway initiating feedback mechanisms to increase pro-inflammatory responses as well as decreasing anti-inflammatory mediators (IL-10). Further work needs to be completed to compare this standard particle to samples collected from various indoor environments in which the occupants vary their activities. Collectively, the results indicate the potential for inhaled air pollution to cause the onset of an inflamed response as well as exacerbating pre-existing immune responses. Furthermore, the data imposes the importance of considering unhealthy individuals when investigating the potential health effects of IAP.

### Supplementary Information

**Supplementary Material 1.**

## Data Availability

The authors declare that the data supporting the findings of this study are available within the paper and its Supplementary Information files. Raw data files are available from the corresponding author upon reasonable request.
