# Supplementary material for: Determining the toxicological effects of indoor air pollution on both a healthy and an inflammatory-comprised model of the alveolar epithelial barrier in vitro
Source: Part Fibre Toxicol. 2024 May 17;21:25. doi: 10.1186/s12989-024-00584-8 (PMC11100169; doi:10.1186/s12989-024-00584-8)
Supplement: Supplementary file 1 — Supplementary Material 1. [file 12989_2024_584_MOESM1_ESM.docx]

Determining the toxicological effects of indoor air pollution on both a healthy and an inflammatory-comprised model of the alveolar epithelial barrier *in vitro*

Kirsty Meldrum^1*^, Stephen J. Evans^1^, Michael J. Burgum^1^, Shareen H. Doak^1^, and Martin J.D. Clift^1*^

^1^ *In Vitro* Toxicology Group, Swansea University Medical School, Swansea University, SA2 8PP, Wales, United Kingdom

^*^Corresponding authors

Dr Kirsty Meldrum; [kirsty.meldrum@swansea.ac.uk](mailto:kirsty.meldrum@swansea.ac.uk)

Prof Martin J D Clift; [m.j.d.clift@swansea.ac.uk](mailto:m.j.d.clift@swansea.ac.uk)

*In Vitro* Toxicology Group, Swansea University Medical School, Swansea University, Singleton Park Campus, Swansea, SA2 8PP, United Kingdom.

Supplementary Section.

Concentrations found within the indoor environment of particulate matter were measured between 10-15µg/m^3^, equating to 46.42-60.82mg/cm^2^. The deposition within the alveolar region was then considered (~10% deposition – 4.64-6.08µg/cm^2^) and then further calculations for experimental depositions and limitations within the Cloud12 VitroCell system. Giving the final concentrations of NIST 2583 applied, which were 608, 464, 232ng/cm^2^.

10-15µg/m^3^

=> 46415µg/cm^2^ – 60822.02 µg/cm^2^

=> 46.415mg/cm^2^  - 60.822mg/cm^2^

10% of this will deposit into the alveolar region of the airways.

=>4.64mg/cm^2^ and 6.08mg/cm^2^

Consider the limitations of the VitroCell Cloud 12 system and the concentration that can be measured by the QCM to give us a range of 5, 10 and 15µg/m^3^

Final concentrations used =

- 232ng/cm^2^
- 464ng/cm^2^
- 608ng/cm^2^

Figure S1. Calculations to determine environmentally relevant concentrations of indoor air pollution particles (specifically NIST 2583). Concentrations were established from indoor environments and measured using real-time sensor measurements before considering alveolar deposition and VitroCell capabilities and limitations.


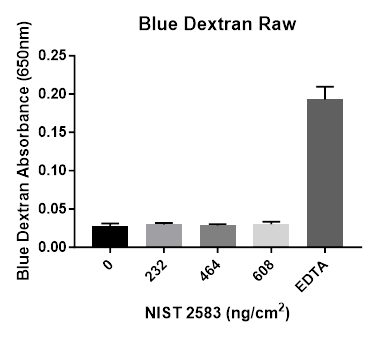

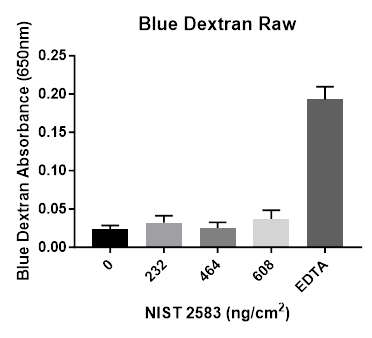

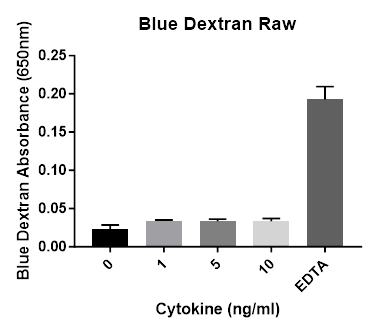


**A B C**

Figure S2. Raw membrane integrity values, 24 hour post exposure of NIST 2583 on the unstimulated (A) A549+dTHP-1 co-culture; 24 hour post exposure of 1, 5, and 10ng/ml of IL-5, IL-13 and IL-4 (B) on the apical side of the stimulated A549+dTHP-1 co-culture; and, 24 hour post exposure of NIST 2583 on an “inflamed” (C) A549+dTHP-1 co-culture. Cells were exposed for 24 hours before analysing membrane integrity (blue dextran). n=3 with all assays performed in triplicate. The data is presented as the raw mean ±Standard deviation.
